# Differences of clinical features and outcomes between male and female elderly patients in gastric cancer

**DOI:** 10.1038/s41598-023-44465-0

**Published:** 2023-10-11

**Authors:** Hiroshi Arakawa, Shuhei Komatsu, Hajime Kamiya, Keiji Nishibeppu, Takuma Ohashi, Hirotaka Konishi, Atsushi Shiozaki, Takeshi Kubota, Hitoshi Fujiwara, Eigo Otsuji

**Affiliations:** Division of Digestive Surgery (Gastric Surgery Division), Department of Surgery, Kyoto Prefectural University of Medicine, 465 Kajii-cho, Kawaramachi-hirokoji, Kamigyo-ku, Kyoto, 602-8566 Japan

**Keywords:** Cancer, Gastroenterology, Risk factors

## Abstract

Although the average life span differs between males and females, little is known about differences in clinical features and short and long-term outcomes between elderly male and female gastric cancer patients. This study was designed to clarify these issues to identify the possibility for sex-based treatment strategies in elderly gastric cancer patients. This study included 295 consecutive elderly gastric cancer patients (75 years or older) who underwent curative gastrectomy between 1997 and 2016. We defined postoperative complications as Clavien–Dindo classification grade II or higher. Comorbidities were present in 67% of all patients. Males tended to have more comorbidities than females (*P* = 0.077). Male patients had significantly more upper gastric cancers (*P* = 0.001), a higher incidence of postoperative complications (*P* = 0.045), and poorer prognoses than females (*P* = 0.003). Multivariate analysis revealed that being male was an independent risk factor for postoperative complications (Odds ratio 2.5, *P* = 0.045) and a poor prognostic factor (Hazard ratio 1.81, *P* = 0.008). Patients who underwent limited surgery without postoperative complications tended to have a better prognosis than patients receiving standard surgery with postoperative complications (3-year overall survival: 78% vs. 55%, *P* = 0.156). Male was an independent risk factor for postoperative complications and an independent poor prognostic factor in elderly gastric cancer patients. To avoid postoperative complications, the limited surgery might be justified for high-risk elderly male patients.

## Introduction

Remarkably, aging societies are increasing worldwide, particularly in developed countries^1–3^. In Japan, the incidence of elderly persons 65 years or older is 28.9%, which is considerably higher than the average incidence of 8.9% globally. Elderly gastric cancer patients are also rising because of an aging society^4^. Elderly patients have frailties across multiple organ systems^5^, which is a risk factor for postoperative complications following gastrectomy^6,7^. Because several studies have already identified that postoperative complications are a poor prognostic factor for patients with gastric cancer^8–11^, establishing appropriate treatment strategies for elderly gastric cancer patients is pivotal to improving short and long-term outcomes.

The average life span differs between male and female elderly persons. Specifically, the average life expectancy of males is 6 years shorter than females (male vs. female: 82 vs. 88 years old) in Japan^12^. However, little is known about differences in clinical features and short and long-term outcomes between males and females following gastrectomy. In this study, we investigated these parameters to clarify these issues.

We hypothesized that the prognosis may be improved by reducing complications in male, which was an independent risk factor for complications. Thus, we proposed that limited surgery could be an effective strategy, especially for patients with comorbidities.

## Materials and methods

### Patients and procedures

The study was institutionally approved by the Kyoto Prefectural University of Medicine (the approved number from the review board, ERB-C-67-5), and each participant provided written informed consent. All methods were performed based on the Declaration of Helsinki. A total of 291 patients aged 75 years or older who underwent curative gastrectomy at Kyoto Prefectural University of Medicine between 1997 and 2016 were included in the study. The clinical characteristics of the patients are shown in Table 1. In this study, we retrospectively analyzed clinicopathological features and early and long-term outcomes.

**Table 1:** Comparison of clinicopathological factors between female and male patients.

	All		Female		Male		*P*-value
	n = 295		n = 114		n = 181		
Age (years)							0.207
≥ 85	34	12%	13	11%	21	12%	
< 85	261	89%	101	89%	160	88%	
BMI (kg/m^2^)							0.766
≥ 25	59	20%	24	21%	35	19%	
< 25	236	80%	90	79%	146	81%	
Histological type							0.003
Undifferentiated	111	38%	55	48%	56	31%	
Differentiated	184	62%	59	52%	125	69%	
Lymphatic invasion							0.233
Positive	142	48%	60	53%	82	45%	
Negative	153	52%	54	47%	99	55%	
Venous invasions							0.473
Positive	160	54%	65	57%	95	53%	
Negative	135	46%	49	43%	86	48%	
Tumor location							0.001
U	80	27%	19	17%	61	34%	
M and L	215	73%	95	83%	120	66%	
Pathological N status							0.011
N0	195	66%	83	73%	112	62%	
N1	41	14%	7	6%	34	19%	
N2	30	10%	14	12%	16	9%	
N3	29	10%	10	9%	19	1%	
Pathological T status							0.476
T1	149	51%	63	55%	86	48%	
T2	41	14%	20	18%	21	12%	
T3	67	23%	14	12%	53	29%	
T4	38	13%	17	15%	21	12%	
Tumor size (mm)							0.358
≥ 60	86	29%	37	33%	49	27%	
< 60	209	71%	77	68%	132	73%	
Surgical approach							0.698
Open	204	69%	77	68%	127	70%	
Laparoscopic	91	31%	37	33%	54	30%	
Surgical procedure							0.027
Total	92	31%	25	22%	67	37%	
Distal	188	64%	83	73%	105	58%	
Proximal	15	5%	6	5%	9	5%	
Complications							0.045
≥ Grade II	45	15%	11	10%	34	19%	
< Grade II	250	85%	103	90%	147	81%	
Comorbidities							0.077
Positive	197	67%	69	61%	128	71%	
Negative	98	33%	45	40%	53	29%	

The postoperative follow-up program at our institution comprises a regular physical examination, laboratory blood tests, and chest X-rays every 3 or 6 months. Endoscopy and ultrasonography, or computed tomography, were performed annually for the first 5 years, if possible. All enrolled patients underwent pathological or macroscopic curative resection (R0). Histological types were classified as differentiated (papillary adenocarcinoma, or moderately or well-differentiated adenocarcinoma) or undifferentiated (poorly differentiated or undifferentiated adenocarcinoma, signet-ring cell carcinoma, or mucinous adenocarcinoma) based on the 15th edition of the Japanese Classification of Gastric Carcinoma^13^. We defined patients with postoperative complications as grade II or higher according to the Clavien–Dindo classification system. Additionally, the comorbidities of these patients were classified based on the American Society of Anesthesiologists (ASA) classification. Specifically, we included those with an ASA grade of III and above, necessitating therapeutic interventions.

### Definition of standard surgery and limited surgery

We defined standard surgery as D1 or D1 + lymphadenectomy for clinical T1 and N0 tumors and D2 or D2 + lymphadenectomy with more advanced tumors. In D2 dissections, peri-gastric lymph nodes and all second-tier lymph nodes were completely retrieved. Combined resection was defined as resection of the other organ involved in direct tumor invasion or concurrent cancer^14,15^. On the other hand, we defined limited surgery as surgery with lesser fields of recommended lymphadenectomy according to gastric cancer treatment guidelines, omitting splenectomy.

### Statistical analysis

The Chi-squared test and Fisher's exact probability test were performed for categorical variables, and Student's *t*-test and Mann–Whitney *U* test for unpaired continuous variables to compare clinicopathological characteristics between comparison groups. Survival curves were estimated using the Kaplan–Meier method, and statistical differences were examined with the log-rank test. Data were stratified for multivariate analysis using backward stepwise Cox regression methods. A P-value < 0.05 was considered significant.

### Approval of the research protocol

This study was approved by the institutional review board of the Kyoto Prefectural University of Medicine.

### Informed consent

Patients’ data were collected with written informed consent, approved by the Kyoto Prefectural University of Medicine.

### Ethics approval

This study was institutionally approved by Kyoto Prefectural University of Medicine.

## Results

### Clinicopathological features of gastric cancer in male and female patients

Comorbidities were present in 67% of all patients. Frequent comorbidities were hypertension (38%), heart disease (21%), diabetes (14%), and respiratory disease (9%) (Supplementary Table S1). Male patients tended to have more comorbidities than females (P = 0.077). Specifically, heart disease (P = 0.106), cerebral disease (P = 0.161), and renal disease (P = 0.161) tended to be higher in male patients. Table 1 shows that male patients had significantly higher incidences of undifferentiated and upper gastric cancers than female patients (P = 0.003, P = 0.001). The incidence of postoperative complications was also higher in male patients than in female patients (P = 0.045). The most common postoperative complications were anastomotic leakage (P = 0.506), pneumonia (P = 0.161), pancreatic fistula (P = 0.158), and intra-abdominal abscess (P = 1.000) (Supplementary Table S2).

### Clinical effect of sex differences and clinicopathological factors on postoperative complications

First, we compared clinicopathological factors between patients with and without postoperative complications. Multivariate analysis using logistic regression revealed that being male [*P* = 0.040, OR (Odds ratio) 2.15 (95% CI 1.03–4.46)] and having open gastrectomy [*P* = 0.021, OR 2.73 (95% CI 1.16–6.39)] were independent risk factors for postoperative complications (Table 2).

**Table 2:** Univariate and multivariate analyses to detect possible risk factors for postoperative complications.

	≥ Grade II		< Grade II		Univariate analysis	Multivariate analysis		
	n = 45		n = 250		*P*-value	OR	95% CI	*P*-value
Gender					0.045	2.15	1.03–4.46	0.040
Male	34	76%	147	59%				
Female	11	24%	103	41%				
Age (years)					0.799			
≥ 85	4	9%	30	12%				
< 85	41	91%	220	88%				
BMI (kg/m^2^)					0.229			
≥ 25	12	27%	47	19%				
< 25	33	73%	203	81%				
Histological type					1			
Undifferentiated	17	38%	94	38%				
Differentiated	28	62%	156	62%				
Lymphatic invasion					0.259			
Positive	18	40%	124	50%				
Negative	27	60%	126	50%				
Venous invasion					0.193			
Positive	20	44%	140	56%				
Negative	25	56%	110	44%				
Tumor location					0.1			
U	17	38%	63	25%				
M and L	28	62%	187	75%				
Pathological N status					0.06			
N3	8	18%	21	8%				
N0–2	37	82%	229	92%				
Pathological T status					0.145			
T4	9	20%	29	12%				
T1–3	36	80%	221	88%				
Tumor size (mm)					0.483			
≥ 60	15	33%	71	28%				
< 60	30	67%	179	72%				
Surgical approach					0.015	2.73	1.16–6.39	0.021
Open	38	84%	166	66%				
Laparoscopic	7	16%	84	34%				
Surgical procedure					0.048			
Total	23	49%	69	28%				
Distal, proximal	22	51%	181	72%				
Comorbidities					1.000			
Positive	35	78%	162	65%				
Negative	10	22%	88	35%				
Extent of lymph node					0.121			
Limited	18	40%	99	40%				
Standard	27	60%	151	60%				

### Prognostic factors of elderly male gastric cancer patients

**Figure 1:**
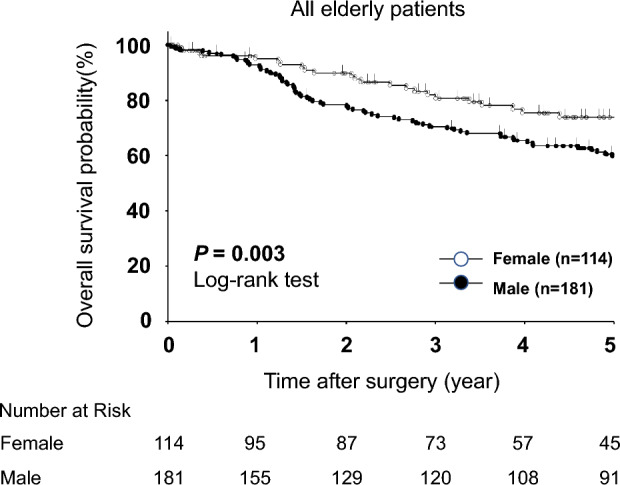
Comparison of overall survival curves between elderly male and female patients. Elderly male patients had poorer prognoses after gastrectomy than female patients (*P* = 0.003).

Next, we investigated the prognostic factors of elderly male gastric cancer patients. In our cohort of 181 elderly male patients with gastric cancer, 36 patients died due to metastasis or recurrence of gastric cancer, 8 patients died from other types of cancer, and 44 patients died from other diseases (Supplementary Table S3). Among these patients, only one patient undergone neoadjuvant chemotherapy (S-1 oral administration). As for adjuvant chemotherapy, only a few patients did receive this treatment: 19 patients were administered S-1 orally, 6 patients received UFT orally, 2 patients were given 5-FU, and 2 other patients received other treatments. Univariate analysis using a log-rank test revealed that elderly male patients had poorer prognoses than female patients after gastrectomy (*P* = 0.003) (Fig. 1). Multivariate analysis using Cox’s proportional hazards model revealed that being male [*P* = 0.008, HR (Hazard ratio) 1.81 (95% CI 1.17–2.80)], elderly [*P* < 0.001, HR 2.79 (95% CI 1.70–4.58)], pN3 [*P* < 0.001, HR 3.03 (95% CI 1.86–4.95)], pT4 [*P* = 0.002, HR 2.11 (95% CI 1.32–3.38)], having open gastrectomy [*P* = 0.004, HR 2.01 (95% CI 1.25–3.23)] and postoperative complications [*P* < 0.001, HR 2.46 (95% CI 1.60–3.77)] were independent poor prognostic factors (Table 3). Regarding male patients, total gastrectomy was an independent poor prognostic factor [*P* = 0.036, OR 2.25 (95% CI 1.06–4.78)], determined using logistic regression (Supplementary Table S4).

**Table 3:** Univariate and multivariate analyses of overall survival after surgery in elderly patients.

	Variables	Univariate analysis	Multivariate analysis		
		*P*-value	HR	95% CI	*P*-value
Gender	Male vs. female	0.003	1.81	1.17–2.80	0.008
Age (years)	≥ 85 vs. < 85	0.002	2.79	1.70–4.58	< 0.001
BMI (kg/m^2^)	≥ 25 vs. < 25	0.765			
Histological type	Undifferentiated vs. differentiated	0.469			
Lymphatic invasion	Positive vs. negative	< 0.001			
Venous invasion	Positive vs. negative	0.001			
Tumor location	U vs. M and L	0.328			
Pathological N status	N3 vs. N0–2	< 0.001	3.03	1.86–4.95	< 0.001
Pathological T status	T4 vs. T1–3	< 0.001	2.11	1.32–3.38	0.002
Tumor size (mm)	≥ 60 vs. < 60	0.004			
Surgical approach	Open vs. laparoscopic	< 0.001	2.01	1.25–3.23	0.004
Surgical procedure	TG vs. DG, PG	0.008			
Complications	≥ Grade II vs. < Grade II	< 0.001	2.46	1.60–3.77	< 0.001
Comorbidities	Present vs. absent	0.870			
Extent of lymph node	Limited vs. standard	0.186			

### Prognostic analysis of male gastric cancer patients considering postoperative complications and type of surgery

**Figure 2:**
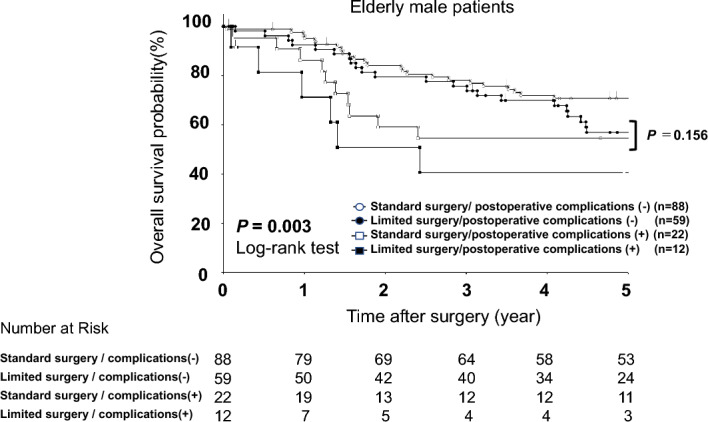
Comparison of overall survival curves with elderly male patients according to the extent of lymphadenectomy and the presence of postoperative complications. Patients who underwent standard surgery had better prognoses than patients who underwent limited surgery (3-year overall survival: 78% vs. 72%, *P* = 0.186). However, patients who underwent limited surgery without developing postoperative complications tended to have a better prognosis than patients receiving standard surgery with postoperative complications (3-year overall survival: 78% vs. 55%, *P* = 0.156).

Finally, we analyzed the prognostic impact of postoperative complications following limited surgery in male patients (standard surgery (T1: n = 50, T2–4: n = 60), limited surgery (T1: n = 36, T2–4: n = 35)). Patients who underwent standard surgery had better prognoses than patients who underwent limited surgery (3-year overall survival: 78% vs. 72%, *P* = 0.186). However, regarding the efficacy of limited surgery to avoid postoperative complications in male patients, patients who underwent limited surgery without postoperative complications tended to have a better prognosis than patients receiving standard surgery who developed postoperative complications (3-year overall survival: 78% vs. 55%, *P* = 0.156) (Fig. 2).

## Discussion

Gastric cancer is among the most common causes of cancer-related death worldwide^16^. Recent advances in diagnostic techniques, minimally invasive surgical methods, and perioperative management have led to the early detection of gastric cancer and declines in mortality and morbidity^17–19^. However, little is known about differences in clinical features and short and long-term outcomes between male and female elderly gastric cancer patients. In this study, we clearly demonstrated that changes associated with advancing age that differ between males and females affect clinical features, postoperative complications, and long-term outcomes in gastric cancer. Namely, being male was an independent risk factor for postoperative complications and an independent poor prognostic factor in elderly gastric cancer patients. Receiving limited surgery without developing postoperative complications contributed to a better prognosis than standard surgery with postoperative complications in male gastric cancer patients. Our results may provide evidence that changes associated with advancing age that differ between elderly male and female gastric cancer patients affect clinical features, postoperative complications, and long-term outcomes and that sex-based treatment strategies might be needed to improve outcomes.

Several studies have examined the relationship between prognosis and sex in various types of cancer, including gastric cancer. Using a large database^20^ in Korea, Huafu et al. reported that male gastric cancer patients had worse prognoses than female patients. On the other hand, Kim et al. reported that males had better prognoses than females in young gastric cancer patients because younger males had a lower incidence of signet ring cell carcinoma^21,22^. In colorectal cancer, Yang et al. conducted a meta-analysis to reveal that males had worse overall survival and cancer-specific survival than females^23^. Previous studies and several meta-analyses in various solid tumors including gastric cancer suggested that males are tended to have postoperative complications more frequently than females as shown in our results^24,25^. Whereas Sah et al. indicated that females were more prone to have postoperative complications following gastric cancer surgery^26,27^. The authors report that females were more prone to serious complications, possibly due to the influence of sex hormones on the overall prognosis. Azzurra et al. suggested the theory that female hormones could maintain the immune tolerance and prevent the excessive inflammatory responses^28^. Thereby, female hormones might potentially contribute to postoperative recovering and reducing the risk of postoperative complications. We also suggest that the hormonal differences between male and female might affect the aging phenomenon and postoperative complications. These issues are currently under evaluation by investigating the various hormone levels, and we will report details in near future.

Another striking finding in this study was that limited surgery without postoperative complications might contribute to a better prognosis than standard surgery with postoperative complications in elderly male patients. Indeed, 3-year overall survival following limited surgery without postoperative complications was higher than for standard surgery with postoperative complications (78% vs. 55% (P = 0.156)) in elderly male patients. Regarding the extent of lymphadenectomy, various studies have shown the efficacy of D2 gastrectomy to be controversial compared to D1 gastrectomy in elderly gastric cancer patients^29–32^. However, two recent studies report that standard D2 surgery contributes to a better prognosis compared to limited surgery, even in elderly gastric cancer patients^33,34^. In real-world data from nationwide general hospitals, patients had more high-risk comorbidities than those in high-volume centers. Therefore, a safer operation strategy might be performing limited surgery to avoid postoperative complications. A recent pivotal study identified that, postoperatively, elderly gastric cancer patients are more likely to die from other diseases rather than from gastric cancer^35^. Moreover, postoperative complications affect the incidence of death from other diseases, especially respiratory disease^36^. Therefore, performing limited surgery for high-risk and/or elderly male patients could be a sex-based treatment strategy for elderly gastric cancer patients.

The European Society for Medical Oncology (ESMO) recommends a geriatric assessment to evaluate functional age for treatment in elderly gastric cancer patients as it is a better predictor of treatment response than chronological age^37^. In the ESMO guideline, limited surgery is already recommended for high-risk patients evaluated using the geriatric assessment. This guideline is very useful considering the aging society globally. On the other hand, a recent Japanese nationwide study proved that the introduction of minimally invasive surgery reduced postoperative complications in high-risk patients, including elderly patients^38^. Indeed, robotic surgery can reduce postoperative complications^39,40^. Therefore, minimally invasive surgery with standard lymphadenectomy may reduce postoperative complications and be an alternative strategy in elderly patients^41^. This issue is also under evaluation at our institute and will be reported in the near future.

A limitation of our study was that the results were retrospectively demonstrated in a small cohort. The long accrual period of this retrospective analysis at a single institute may have incorporated variations in treatment strategies. Therefore, a prospective observational study using several large cohorts or a nationwide clinical database study may be needed to validate our finding that being male is a poor prognostic factor and proposal for a sex-based surgical strategy. Nevertheless, elderly male patients had higher frequencies of comorbidities and postoperative complications. They should be specifically targeted in an effort to improve prognosis by considering comorbidities and performing limited surgery to avoid complications. Also, minimally invasive surgery may be the future strategy for avoiding postoperative complications and improving prognosis. The findings of our research are awaiting confirmation through a prospective trial.

### Supplementary Information

Supplementary Table S1.

Supplementary Table S2.

Supplementary Table S3.

Supplementary Table S4.

## Data Availability

The datasets generated and/or analysed during the current study are not publicly available due to the personal information protection law in Japan but are available after the permission from the institutional review board and the corresponding author on reasonable request.
