# Author Correction: Differences of clinical features and outcomes between male and female elderly patients in gastric cancer

**DOI:** 10.1038/s41598-024-51810-4

**Published:** 2024-01-23

**Authors:** Hiroshi Arakawa, Shuhei Komatsu, Hajime Kamiya, Keiji Nishibeppu, Takuma Ohashi, Hirotaka Konishi, Atsushi Shiozaki, Takeshi Kubota, Hitoshi Fujiwara, Eigo Otsuji

**Affiliations:** Division of Digestive Surgery (Gastric Surgery Division), Department of Surgery, Kyoto Prefectural University of Medicine, 465 Kajii‑cho, Kawaramachi‑hirokoji, Kamigyo‑ku, Kyoto, 602‑8566 Japan

Correction to: *Scientific Reports* 10.1038/s41598-023-44465-0, published online 11 October 2023

The original PDF version of this Article contained an error where sentences were duplicated in the section “Discussion”.

The below sentences have now been removed:

“Our results may provide evidence that changes associated with advancing age that differ between elderly male and female gastric cancer patients affect clinical features, postoperative complications, and long-term outcomes and that sex-based treatment strategies might be needed to improve outcomes.”

“In this study, we clearly identified that being male was an independent risk factor for postoperative complications and an independent poor prognostic factor, particularly in elderly gastric cancer patients. These findings are novel in elderly gastric cancer patients and provide pivotal clinical evidence that changes due to advancing age in elderly male patients negatively affect early and long-term outcomes in gastric cancer.”

The original article is now corrected.

