# Supplementary material for: Differences of clinical features and outcomes between male and female elderly patients in gastric cancer
Source: Sci Rep. 2023 Oct 11;13:17192. doi: 10.1038/s41598-023-44465-0 (PMC10567739; doi:10.1038/s41598-023-44465-0)
Supplement: Supplementary file 1 — Supplementary Table S1. [file 41598_2023_44465_MOESM1_ESM.docx]

**Supplementary table S1: Comparison of comorbidities between female and male patients**

|  | **All** | | **Female** | | **Male** | |  |
| --- | --- | --- | --- | --- | --- | --- | --- |
|  | **n=295** | | **n=114** | | **n=181** | | ***P*-value** |
| **Hypertension** |  |  |  |  |  |  | **0.803** |
| **Positive** | **104** | **35%** | **39** | **34%** | **65** | **36%** |  |
| **Negative** | **191** | **65%** | **75** | **66%** | **126** | **64%** |  |
| **Heart disease** |  |  |  |  |  |  | **0.106** |
| **Positive** | **62** | **21%** | **18** | **16%** | **44** | **24%** |  |
| **Negative** | **234** | **79%** | **96** | **84%** | **137** | **76%** |  |
| **Diabetes** |  |  |  |  |  |  | **0.227** |
| **Positive** | **41** | **14%** | **12** | **11%** | **29** | **16%** |  |
| **Negative** | **254** | **86%** | **102** | **90%** | **152** | **84%** |  |
| **Hepatic disease** |  |  |  |  |  |  | **0.577** |
| **Positive** | **14** | **5%** | **4** | **4%** | **10** | **6%** |  |
| **Negative** | **281** | **95%** | **110** | **97%** | **171** | **95%** |  |
| **Metabolic disorder** |  |  |  |  |  |  | **0.630** |
| **Positive** | **19** | **6%** | **6** | **5%** | **13** | **7%** |  |
| **Negative** | **276** | **94%** | **108** | **95%** | **168** | **93%** |  |
| **Respiratory disease** |  |  |  |  |  |  | **0.290** |
| **Positive** | **25** | **9%** | **7** | **6%** | **18** | **10%** |  |
| **Negative** | **270** | **92%** | **107** | **94%** | **163** | **90%** |  |
| **Cerebral disease** |  |  |  |  |  |  | **0.161** |
| **Positive** | **9** | **3%** | **1** | **1%** | **8** | **4%** |  |
| **Negative** | **286** | **97%** | **113** | **90%** | **173** | **96%** |  |
| **Renal disease** |  |  |  |  |  |  | **0.161** |
| **Positive** | **4** | **1%** | **0** | **0%** | **4** | **2%** |  |
| **Negative** | **291** | **99%** | **114** | **100%** | **177** | **98%** |  |
| **Autoimmune disease** |  |  |  |  |  |  | **0.210** |
| **Positive** | **6** | **2%** | **4** | **4%** | **2** | **1%** |  |
| **Negative** | **289** | **98%** | **110** | **97%** | **179** | **99%** |  |
| **Endocrine disease** |  |  |  |  |  |  | **1.000** |
| **Positive** | **5** | **2%** | **2** | **2%** | **3** | **2%** |  |
| **Negative** | **290** | **98%** | **112** | **98%** | **178** | **98%** |  |
