# Supplementary material for: Differences of clinical features and outcomes between male and female elderly patients in gastric cancer
Source: Sci Rep. 2023 Oct 11;13:17192. doi: 10.1038/s41598-023-44465-0 (PMC10567739; doi:10.1038/s41598-023-44465-0)
Supplement: Supplementary file 2 — Supplementary Table S2. [file 41598_2023_44465_MOESM2_ESM.docx]

**Supplementary table S2: Complications after gastrectomy in elderly patients**

|  | **All** | | **Female** | | **Male** | |  |
| --- | --- | --- | --- | --- | --- | --- | --- |
|  | **n=295** | | **n=114** | | **n=181** | | ***P*-value** |
| **Anastomotic leakage** | **23** | **7.8%** | **7** | **6.1%** | **16** | **8.8%** | **0.506** |
| **Pneumonia** | **9** | **3.1%** | **1** | **0.9%** | **8** | **4.4%** | **0.161** |
| **Pancreatic fistula** | **8** | **2.7%** | **1** | **0.9%** | **7** | **3.9%** | **0.158** |
| **Intra-abdominal abscess** | **8** | **2.7%** | **3** | **2.6%** | **5** | **2.8%** | **1.000** |
| **All** | **45** | **15.3%** | **11** | **9.6%** | **34** | **18.8%** | **0.045** |
