# Supplementary material for: Differences of clinical features and outcomes between male and female elderly patients in gastric cancer
Source: Sci Rep. 2023 Oct 11;13:17192. doi: 10.1038/s41598-023-44465-0 (PMC10567739; doi:10.1038/s41598-023-44465-0)
Supplement: Supplementary file 3 — Supplementary Table S3. [file 41598_2023_44465_MOESM3_ESM.docx]

**Supplementary table S3: Cause of death after gastrectomy in elderly male patients**

|  | **n=181** | |
| --- | --- | --- |
| **Metastasis or recurrence of gastric cancer** | **36** | **19.9%** |
| **Other types of cancer** | **8** | **4.4%** |
| **Other diseases** | **44** | **24.3%** |
| **All** | **88** | **48.6%** |
