# Supplementary material for: Differences of clinical features and outcomes between male and female elderly patients in gastric cancer
Source: Sci Rep. 2023 Oct 11;13:17192. doi: 10.1038/s41598-023-44465-0 (PMC10567739; doi:10.1038/s41598-023-44465-0)
Supplement: Supplementary file 4 — Supplementary Table S4. [file 41598_2023_44465_MOESM4_ESM.docx]

**Supplementary table S4: Univariate analyses to risk factors for postoperative complication in elder male patients**

|  | **> Grade II** | | **< Grade II** | | **Univariate** | **Multivariate analysis** | | | | |
| --- | --- | --- | --- | --- | --- | --- | --- | --- | --- | --- |
|  | **n=34** | | **n=147** | | ***P*-value** | **OR** | **95%CI** | | | ***P*-value** |
| **Age(years)** |  |  |  |  |  |  |  |  |  |  |
| **> 85** | **4** | **12%** | **17** | **12%** | **1.000** |  |  |  |  |  |
| **< 85** | **30** | **88%** | **130** | **88%** |  |  |  |  |  |  |
| **BMI(kg/m²)** |  |  |  |  |  |  |  |  |  |  |
| **> 25** | **10** | **29%** | **25** | **17%** | **0.146** |  |  |  |  |  |
| **< 25** | **24** | **71%** | **122** | **83%** |  |  |  |  |  |  |
| **Histological type** |  |  |  |  |  |  |  |  |  |  |
| **Undifferentiated** | **12** | **35%** | **44** | **30%** | **0.543** |  |  |  |  |  |
| **Differentiated** | **22** | **65%** | **103** | **70%** |  |  |  |  |  |  |
| **Lymphatic invasion** |  |  |  |  |  |  |  |  |  |  |
| **Positive** | **13** | **38%** | **69** | **47%** | **0.445** |  |  |  |  |  |
| **Negative** | **21** | **62%** | **78** | **53%** |  |  |  |  |  |  |
| **Venous invasions** |  |  |  |  |  |  |  |  |  |  |
| **Positive** | **15** | **44%** | **80** | **54%** | **0.342** |  |  |  |  |  |
| **Negative** | **19** | **56%** | **67** | **46%** |  |  |  |  |  |  |
| **Tumor location** |  |  |  |  |  |  |  |  |  |  |
| **U** | **12** | **37%** | **49** | **33%** | **0.842** |  |  |  |  |  |
| **M and L** | **22** | **60%** | **98** | **67%** |  |  |  |  |  |  |
| **Pathological N status** |  |  |  |  |  |  |  |  |  |  |
| **N3** | **5** | **15%** | **14** | **10%** | **0.361** |  |  |  |  |  |
| **N0-2** | **29** | **85%** | **133** | **91%** |  |  |  |  |  |  |
| **Pathological T status** |  |  |  |  |  |  |  |  |  |  |
| **T4** | **6** | **18%** | **15** | **10%** | **0.238** |  |  |  |  |  |
| **T1-3** | **28** | **82%** | **132** | **90%** |  |  |  |  |  |  |
| **Tumor size(mm)** |  |  |  |  |  |  |  |  |  |  |
| **> 60** | **10** | **29%** | **39** | **27%** | **0.831** |  |  |  |  |  |
| **< 60** | **24** | **71%** | **108** | **74%** |  |  |  |  |  |  |
| **Surgical approach** |  |  |  |  |  |  |  |  |  |  |
| **Open** | **28** | **82%** | **99** | **67%** | **0.099** |  |  |  |  |  |
| **Laparoscopic** | **6** | **18%** | **48** | **33%** |  |  |  |  |  |  |
| **Surgical procedure** |  |  |  |  |  | **2.25** | **1.06** | **-** | **4.78** | **0.036** |
| **Total** | **18** | **53%** | **49** | **33%** | **0.048** |  |  |  |  |  |
| **Distal, Proximal** | **16** | **47%** | **98** | **67%** |  |  |  |  |  |  |
| **Comorbidities** |  |  |  |  |  |  |  |  |  |  |
| **Positive** | **28** | **82%** | **100** | **32%** | **0.322** |  |  |  |  |  |
| **Negative** | **6** | **18%** | **47** | **68%** |  |  |  |  |  |  |
| **Extent of lymph node** |  |  |  |  |  |  |  |  |  |  |
| **Limited** | **12** | **35%** | **59** | **40%** | **0.698** |  |  |  |  |  |
| **Standard** | **22** | **65%** | **88** | **60%** |  |  |  |  |  |  |
